# Erratum

**DOI:** 10.1111/hex.12882

**Published:** 2019-05-31

In this paper^1^, the references cited in Tables 3 and 4 have been renumbered to correspond with the correct list of references in the “Reference” section.

**Table 3:** Classification of information needs in mental disorders identified in n=8 included articles

Category (overall %, schizophrenia %, depression %)^a^	Subcategory^b^	% of articles^c^	References
Basic facts (34.7%, 30.6%, 40.8%)		100.0%, n* *= 8	
	Personal experiences of other people with mental disorders (16.7%)		34,41,43,44,53
	Early warning signs and relapse (16.7%)		34,42,43,45
	Symptoms (16.7%)		34,42,45,49
	Diagnostics (11.9%)		34,41,44,49
	Causal model/vulnerability (9.5%)		34,41,49,53
	Course of disease and prognosis (9.5%)		45,49,53
	Recent research (7.1%)		34
	Prevalence (4.8%)		34,49
	Normal or mentally ill (4.8%)		49
	Comorbid mental health problems (2.4%)		41
Treatment (21.5%, 20.8%, 22.4%)		100.0%, n = 8	
	Side effects of medications (46.2%)		34,41‐45,49
	Psychiatric medications (30.8%)		34,41,44,45,49
	Treatment options (11.5%)		41,49,53
	Psychosocial treatment/psychotherapy (11.5%)		45,49
Coping (19.0%, 19.4%, 18.4%)		87.5%, n = 7	
	Coping with symptoms (26.1%)		41‐43,45,49,53
	Strategies for solving problems (21.7%)		34,42,43
	Stress management (21.7%)		34,43
	Coping with stigma (13.0%)		34,41,45
	Recovery (13.0%)		41,49
	Coping with anger, violence, assaultive behaviour (4.3%)		43
Medical system (7.4%, 5.6%, 10.2%)		62.5%, n = 5	
	Access to services and professionals (66.7%)		34,41‐43,49
	Knowledge of health professionals (22.2%)		41,49
	Mental health‐care system (11.1%)		41,49
Working and living conditions (6.6%, 8.3%, 4.1%)		50.0 %, n = 4	
	Legal rights (37.5%)		41,45
	Financial assistance (25.0%)		49
	What happens when parent dies (25.0%)		34
	Roadworthiness (12.5%)		49
Enhancing social functioning (5.8%, 8.3%, 2.0%)		50.0 %, n = 4	
	Communication with relatives (28.6%)		42,43
	Social relationships (28.6%)		34,42
	Leisure and recreation activities (14.3%)		34
	Independent living skills (14.3%)		42
	Setting limits on the patients’ behaviour (14.3%)		34
Self‐help and peer support (5.0%, 6.9%, 2.0%)		50.0 %, n = 4	45
	Patients’ self‐help organizations (50.0%)		34,42,45
	Relatives’ self‐help organizations and support (33.3%)		44
	Useful books (16.7%)		44

a Relative frequency of identified needs coded within each of the seven main categories determined by dividing the sum of identified units of needs coded within each main category by the 121 units of identified units of needs within the entire body of included articles.

b Subcategories are ordered from top to bottom related to their relative frequency by dividing the sum of identified units of needs coded within each of the 35 subcategories by the sum of all identified units of needs coded within the respective main category. In the case of identical frequencies, subcategories are ordered alphabetically.

c Number and relative frequency of articles in which each main category was coded.

**Table 4:** Classification of treatment decisions in mental disorders identified in n = 4 included studies on decision‐making needs

Category (overall %, schizophrenia %, depression %)^a^	Subcategory^b^	% of articles^c^	References
Medication treatment (23.2%, 21.4%, 25.0%)	Psychiatric medication (30.8%)	100%, n = 4	36,37,46,48
	Change medication (15.4%)		36,37
	Dosage (15.4%)		37,48
	Addition of antidepressant medication (7.7%)		48
	Application mode, that is depot or oral (7.7%)		48
	Choice of antipsychotic agent (7.7%)		48
	Discontinue medication (7.7%)		37
	Tapering‐off medication (7.7%)		36
Treatment setting (25.0%, 25.0%, 25.0%)	Inpatient treatment (21.4%)	100%, n = 4	36,37,48
	Discharge/leave hospital (14.3%)		46,48
	From who to seek help (14.3%)		36,37
	Absence from the ward (7.1%)		48
	Attend a day programme (7.1%)		37
	Change in treatment setting (7.1%)		46
	Find support in vs. outside neighbourhood (7.1%)		37
	Open vs. closed ward (7.1%)		48
	Relocate to get specialized treatment (7.1%)		37
	Treatment after discharge (7.1%)		46
General treatment issues (14.3%, 21.4%, 7.1%)	Diagnostic examinations (25.0%)	100%, n = 4	46,48
	Disciplinary measures/Physical and social restraints (25.0%)		46,48
	Frequency of appointments (25.0%)		36,37
	Participation of relatives (12.5%)		48
	Regular drug screens (12.5%)		48
Non‐medication treatment (14.3%, 14.3%, 14.3%)4	Psychotherapy (50.0%)	100%, n = 4	36,37,46,48
	Attend counselling (12.5%)		37
	Electroconvulsive therapy (12.5%)		37
	Psychoeducation (12.5%)		48
	Work therapy (12.5%)		48
Working and living conditions (14.3%, 17.9%, 10.7%)	Employment and education (50.0%)	100%, n = 4	36,37,46,48
	Live arrangements, for example group home, living on own (37.5%)		37,46,48
	Legal guardianship (12.5%)		48
Lifestyle (8.9%, 0.0%, 17.9%)	Give up alcohol (20.0%)	25%, n = 1	37
	Improve communication skills (20.0%)		37
	Join a support group (20.0%)		37
	Keep an active lifestyle (20.0%)		37
	Participate in social activities (20.0%)		37

a Relative frequency of decisions coded within each of the six main categories determined by dividing the sum of identified decisions coded within each main category by the 56 decisions identified within the entire body of included articles.

b Subcategories are ordered from top to bottom related to their relative frequency by dividing the sum of identified decisions coded within each of the 36 subcategories by the sum of all identified decisions coded within the respective main category. In the case of identical frequencies, subcategories are ordered alphabetically.

c Number and relative frequency of articles in which each main category was coded.

Here are Tables 3 and 4 with modified reference citations:

We apologize for these errors.
